# Mitochondria-targeted nanotechnology in cardiovascular diseases: a review of recent advances

**DOI:** 10.1093/rb/rbag040

**Published:** 2026-03-09

**Authors:** Sijia Sun, Manxiang Wu, Pengli Zhang, Linglin Sun, Wenjing Zhong, Sisi Zheng, Yanjin Li, Jianbin Li, Qiang Li

**Affiliations:** Department of Radiology, The First Affiliated Hospital of Zhejiang Chinese Medical University, Zhejiang Provincial Hospital of Chinese Medicine, Hangzhou 310006, China; Department of Radiology, The First Affiliated Hospital of Zhejiang Chinese Medical University, Zhejiang Provincial Hospital of Chinese Medicine, Hangzhou 310006, China; Department of Radiology, The Affiliated People’s Hospital of Ningbo University, Ningbo 315211, China; Department of Radiology, The First Affiliated Hospital of Zhejiang Chinese Medical University, Zhejiang Provincial Hospital of Chinese Medicine, Hangzhou 310006, China; Department of Radiology, The First Affiliated Hospital of Zhejiang Chinese Medical University, Zhejiang Provincial Hospital of Chinese Medicine, Hangzhou 310006, China; Department of Radiology, The First Affiliated Hospital of Zhejiang Chinese Medical University, Zhejiang Provincial Hospital of Chinese Medicine, Hangzhou 310006, China; Department of Radiology, The First Affiliated Hospital of Zhejiang Chinese Medical University, Zhejiang Provincial Hospital of Chinese Medicine, Hangzhou 310006, China; Department of Radiology, The Affiliated People’s Hospital of Ningbo University, Ningbo 315211, China; Department of Radiology, The First Affiliated Hospital of Zhejiang Chinese Medical University, Zhejiang Provincial Hospital of Chinese Medicine, Hangzhou 310006, China

**Keywords:** cardiovascular diseases, mitochondria-targeted nanotechnology, design strategy, delivery efficiency

## Abstract

Cardiovascular diseases (CVDs) remain the leading cause of global mortality, with mitochondrial dysfunction serving as a central pathological hub in conditions such as atherosclerosis, myocardial ischemia-reperfusion injury and heart failure. Current mitochondrial-regulating drugs are severely limited by low bioavailability, short duration of action, poor targeting specificity and off-target effects, highlighting an urgent need for precise delivery systems. Nanocarriers, with tunable physicochemical properties and surface functionalization potential, enable hierarchical targeting of diseased cardiac tissues and mitochondria, offering a novel solution to overcome these limitations. Preclinical models have shown promising efficacy, particularly in alleviating oxidative stress damage in ischemic cardiomyopathy, improving energy metabolism in heart failure and promoting tissue repair. These encouraging results have sparked growing interest in the application of nanomaterials for mitochondrial-targeted diagnosis and treatment of CVDs. This review first outlines the role of mitochondrial dysfunction in CVD pathogenesis, covering impaired oxidative phosphorylation, excessive reactive oxygen species production, disrupted mitochondrial dynamics and defective mitophagy. It, then, focuses on the design strategies of nanotherapeutics based on a hierarchical targeting concept, encompassing the selection of biocompatible carriers, optimization of size and morphology, tissue or cell-specific targeting modifications, mitochondrial ligand modifications, as well as the loading and therapeutic mechanisms of various therapeutic agents. Furthermore, it provides an in-depth analysis of key physiological barriers such as hemodynamic shear stress, endothelial barrier and extracellular matrix hindrance, along with intracellular trafficking challenges including lysosomal escape and immune clearance, which all impact delivery efficiency. This review aims to offer insights to advance the rational development and clinical translation of mitochondria-targeted nanomedicines for CVDs.

## Graphical Abstract

**Graphical Abstract:**
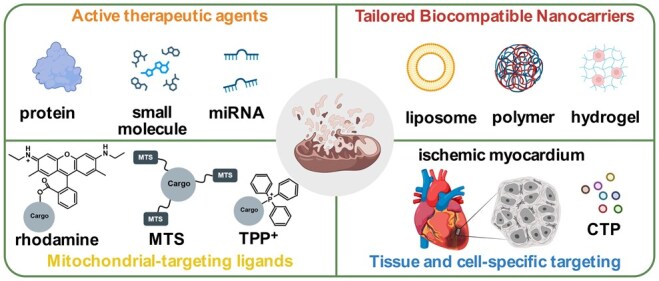

## Introduction

Cardiovascular diseases (CVDs) remain the leading cause of death globally, accounting for over 18 million deaths annually. The increasing burden of an aging population and metabolic diseases continues to drive up the global incidence and mortality of CVDs, posing severe socioeconomic challenges [1, 2]. Although revascularization techniques like percutaneous coronary intervention and coronary artery bypass grafting have achieved significant clinical success, they primarily address structural vascular occlusions. However, these interventions cannot reverse progressive cardiomyocyte loss or inhibit pathological cardiac remodeling, often failing to prevent the eventual progression to heart failure [3]. The extremely limited regenerative capacity of adult cardiomyocytes, coupled with post-injury fibrotic scar formation that further impairs cardiac function, underscores the critical need for novel therapies targeting the molecular mechanisms driving cardiac deterioration [4]. The heart is one of the most energy-demanding organs, and its functional integrity heavily relies on mitochondrial structure and function. Substantial evidence indicates that mitochondrial dysfunction plays a central role in the pathogenesis of various CVDs [5–8]. Impairments in mitochondrial oxidative phosphorylation, excessive reactive oxygen species (ROS) production, defective autophagy and imbalances in mitochondrial dynamics can directly lead to cardiomyocyte death, interstitial fibrosis and maladaptive ventricular remodeling [5–7]. Several drugs aimed at improving mitochondrial function have been developed, such as CoQ10, Mitoquinone (MitoQ), Decyl triphenylphosphonium (SkQ1), Mdivi-1 and P110. However, their clinical application is limited by issues like low bioavailability, short duration, off-target effects and dependency on specific molecular efficacies, which restricts their ability to precisely modulate mitochondrial pathways [9–12].

Emerging drug delivery carriers, such as MITO-Porter and ROS-responsive carriers, have demonstrated enhanced efficacy in restoring mitochondrial integrity and improving cardiac function [13, 14]. Ingeniously designed nanocarriers can not only significantly improve drug stability and bioavailability but also, through targeted modifications, enable precise delivery of therapeutics to mitochondria within diseased tissues/cells, thereby translating the potential concept of mitochondrial modulation into practical and effective therapeutic interventions. However, the cardiovascular system presents several physiological features that are challenging for nanomedicine delivery. For instance, high-velocity blood flow and shear stress result in short nanoparticle retention times and facilitate rapid clearance; cardiomyocytes are terminally differentiated with limited repair capacity post-injury, narrowing the therapeutic window; and the dense, complex cardiac vascular network requires nanoparticles to overcome multiple biological barriers, including endothelial permeability and lysosomal degradation, for effective accumulation at target sites [15, 16]. These factors collectively dictate that the design of mitochondria-targeted nanomedicines for CVDs must carefully consider the unique physiological and pathological context. Furthermore, existing reviews typically focus on mitochondrial therapy or nanomedicine delivery in isolation, lacking an integrated discussion of hierarchical targeting strategies and cardiovascular-specific physiological barriers. This gap underscores the necessity of the current review, which aims to bridge these components, provide a holistic perspective and offer a systematic discussion of the delivery mechanism, targeting efficiency and clinical translation of mitochondria-targeted nanocarriers. Therefore, a systematic discussion on the delivery mechanisms, targeting efficiency and clinical translation of mitochondria-targeted nanocarriers for CVDs is warranted. Against this background, this review systematically examines the central role of mitochondrial dysfunction in various CVDs, summarizes the design strategies and preclinical/clinical progress of mitochondria-targeted nanomedicines for CVDs, and analyzes the key factors limiting effective nanomedicine delivery within the cardiovascular system along with potential solutions. Additionally, we outline the core challenges facing clinical translation in this field and provide perspectives on future directions. This review aims to serve as a theoretical reference for advancing the rational development and translation of mitochondria-targeted nanomedicines for CVDs.

## Mitochondria: a key hub in CVD pathogenesis

### Overview of mitochondrial structure and function

**Figure 1:**
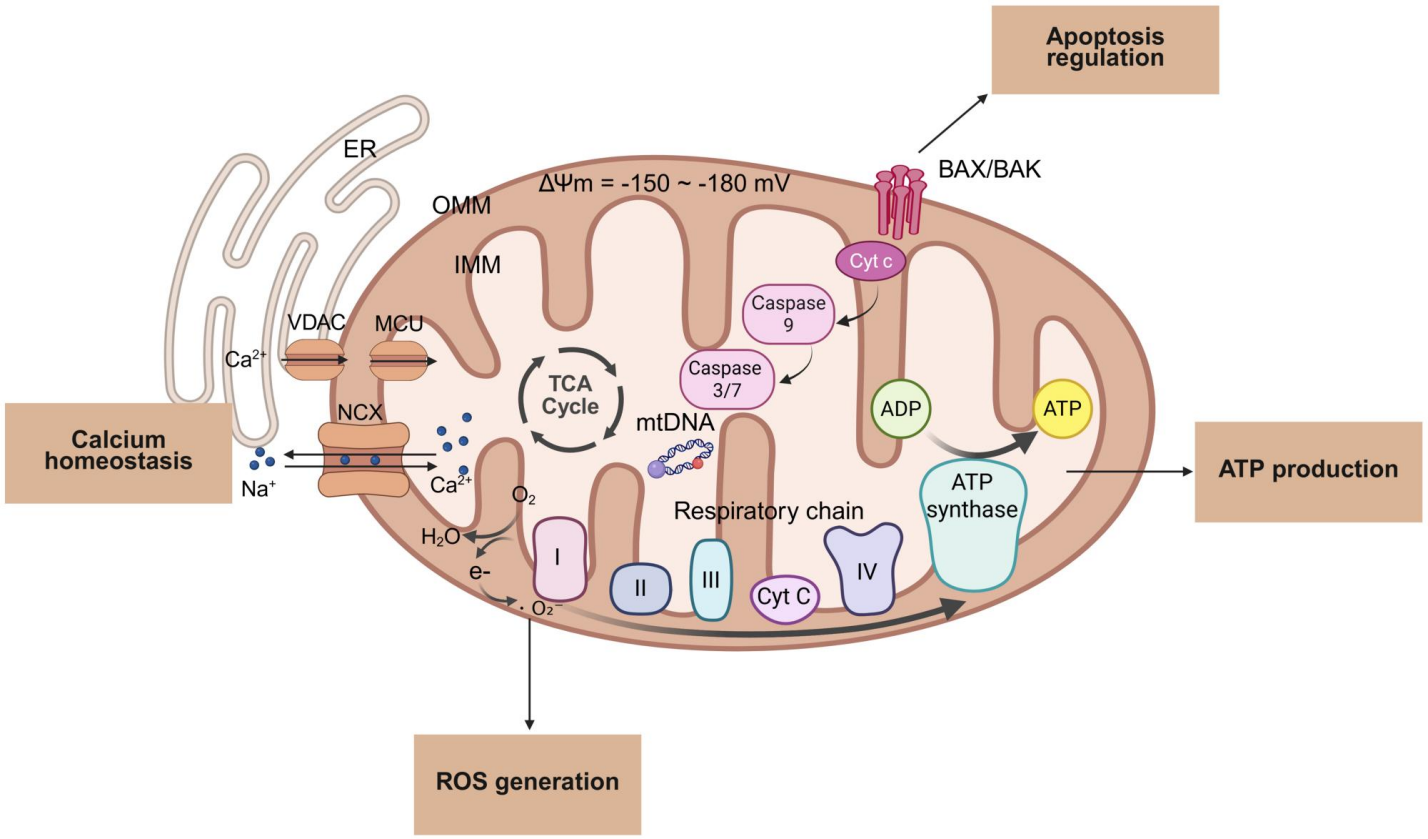
Schematic diagram of the main structure and function of mitochondria. Mitochondria participate in various biological processes through their precise structural components, primarily including metabolism and energy generation, redox homeostasis, substance transport and regulation of apoptosis. ER, endoplasmic reticulum; OMM, outer mitochondrial membrane; IMM, inner mitochondrial membrane; ΔΨm, mitochondrial membrane potential; MCU, mitochondrial calcium uniporter; NCX, Na^+^/Ca^2+^ exchanger; TCA cycle, tricarboxylic acid cycle; Cyt c, cytochrome c; Caspase 9, cysteine-aspartic protease 9. (Image created with BioRender.com, with permission).

Mitochondria are essential organelles in eukaryotic cells, comprising four main compartments: the outer membrane, inner membrane, intermembrane space and matrix. The outer membrane contains porin channels that allow permeability to small molecules; among these, the BAX/BAK pores regulate mitochondrial membrane permeability and facilitate the release of factors like cytochrome c during apoptosis, activating downstream signals such as caspase-9 [17]. The inner membrane is highly invaginated, forming cristae that vastly increase its surface area. Embedded within the cristae are the electron transport chain complexes (I-IV) and ATP synthase (Complex V), which together constitute the oxidative phosphorylation (OXPHOS) system. This system is responsible for establishing and maintaining the mitochondrial membrane potential (ΔΨm = 150–180 mV) and driving ATP synthesis [18]. During electron transfer, a small fraction of electrons leaks prematurely from Complexes I and III, reacting with oxygen to generate superoxide anions, the primary source of mitochondrial ROS. The matrix contains enzymes for the tricarboxylic acid (TCA) cycle and circular mitochondrial DNA (mtDNA), which encodes some respiratory chain proteins, collectively supporting energy metabolism [18]. Furthermore, mitochondria closely interact with the endoplasmic reticulum (ER) to regulate and maintain cellular calcium homeostasis. The ER forms membrane contact sites with mitochondria, allowing efficient calcium transfer from the ER to the mitochondrial intermembrane space. The mitochondrial calcium uniporter (MCU), located on the inner membrane, mediates calcium influx into the matrix, elevating matrix calcium levels to activate key metabolic enzymes. Conversely, the sodium-calcium exchanger (NCX) participates in calcium efflux, maintaining dynamic balance. Increased matrix calcium concentration can enhance energy output by activating metabolic enzymes; however, calcium overload can exacerbate electron leakage, promote ROS generation and subsequently trigger apoptotic pathways [19] (Figure 1).

### Mitochondrial dysfunction and CVDs

#### Atherosclerosis

Atherosclerosis (AS) is a chronic inflammatory disease driven by endothelial injury, lipid deposition and oxidative stress [20]. Within the pathological microenvironment of AS, mtDNA is particularly vulnerable to oxidative damage, deletion and copy number abnormalities due to its lack of histone protection, proximity to ROS generation sites and limited repair capacity. Notably, although mtDNA damage is often associated with oxidative stress, studies within AS plaques suggest that the primary consequence of mtDNA abnormalities is not necessarily a marked increase in ROS levels, but rather insufficient ATP synthesis, exacerbated apoptosis and abundant secretion of pro-inflammatory cytokines [21, 22]. This finding implies that the mechanism by which mtDNA abnormalities drive AS may lean more towards inducing an energy crisis and directly activating inflammatory signaling. Given the limited efficacy of antioxidant therapies in AS, developing drugs that regulate mtDNA replication and synthesis or delivering gene-editing tools to correct mtDNA defects, may represent more promising new directions for AS treatment [23–25].

#### Ischemia-reperfusion injury

Myocardial ischemia-reperfusion injury (MIRI) is characterized by exacerbated myocardial damage following the restoration of blood flow post-ischemia, with mitochondrial dysfunction recognized as a central driver of its pathophysiological process [26]. During the ischemic phase, cardiomyocytes experience energy depletion; upon reperfusion, this is further compounded by oxidative stress and calcium dysregulation, which collectively induce cardiomyocyte death [27–30]. Targeted modulation of mitochondrial fission, enhancement of antioxidant defenses and normalization of calcium handling have been shown to mitigate MIRI effectively [29, 31–33]. Notably, autophagy requires careful regulation due to its context-dependent effects—while beneficial early in reperfusion by removing damaged mitochondria, its overactivation through pathways such as PINK1-Parkin can exacerbate damage [27]. Furthermore, released mtDNA activates the cGAS-STING pathway, amplifying inflammatory injury [34]. Thus, an integrated therapeutic approach should aim to appropriately time autophagy modulation while concurrently targeting oxidative stress and inflammation [35].

#### Heart failure

Under normal conditions, the heart relies primarily on OXPHOS for energy, with fatty acid and glucose oxidation serving as the main ATP sources, supplemented by lactate, ketones and branched-chain amino acids. In heart failure (HF), this efficient metabolic profile shifts toward a less efficient dependence on glycolysis and ketone oxidation, a change closely linked to mitochondrial dysfunction [36]. mtDNA mutations and copy number reductions, caused by aging, epigenetic changes or various causes of HF, compromise the integrity of the respiratory chain and reduce oxidative phosphorylation efficiency [37]. In addition, disrupted mitochondrial dynamics can also lead to network fragmentation, reducing respiratory chain assembly sites and weakening the coordination between substrate metabolism and energy conversion [37]. These mitochondrial damages culminate in an energy crisis during disease progression. Therefore, targeting mitochondrial dysfunction and correcting the resulting metabolic imbalance is an important therapeutic strategy to improve cardiac function in HF.

#### Other cardiovascular diseases

Mitochondrial dysfunction also plays a significant role in the pathophysiology of other CVDs. For instance, doxorubicin (DOX)-induced cardiotoxicity involves multiple processes, including mitochondrial oxidative stress, OXPHOS disruption and mitochondrial permeability transition. DOX can directly interfere with the redox cycling of mitochondrial complex I or bind to cardiolipin, causing energy metabolism disorders. It can also indirectly damage mitochondria via nuclear-mediated topoisomerase IIβ inhibition, often accompanied by abnormal mitophagy flux. Furthermore, DOX can induce “mitochondrial memory” through selective oxidation of mtDNA and epigenetic changes. This leads to cumulative, dose-dependent toxicity that persists long-term, explaining the susceptibility to delayed cardiomyopathy and the differences in toxicity profiles between children and adults [38]. Recent studies have also revealed widespread mitochondrial dysfunction in hereditary thoracic aortic aneurysms and abdominal aortic aneurysms. Patients’ vascular smooth muscle cells commonly exhibit mtDNA abnormalities, including copy number depletion, heteroplasmic insertions, structural rearrangements and mutation enrichment in the D-loop region [39, 40]. Nicotinamide adenine dinucleotide (NAD+) in mitochondria participates in regulating mtDNA replication and repair. Supplementing NAD+ precursors or maintaining sufficient intracellular NAD+ levels has been shown to promote proline biosynthesis, thereby inhibiting aneurysm progression [41]. Additionally, mitochondrial structural and functional disturbances are closely associated with the pathological processes in disease models such as septic cardiomyopathy and diabetic cardiomyopathy [42, 43].

## Design strategies for mitochondria-targeted nanomedicines

Given the central role of mitochondria in CVDs and their significant potential as therapeutic targets, developing nanomedicines capable of precisely regulating mitochondrial function has become a crucial research direction. Successful mitochondria-targeted nanocarriers for CVDs should adhere to the design principle of hierarchical targeting, which relies on a series of precisely controlled delivery steps across spatial scales [44].

### Nanocarriers with good biocompatibility

Various materials with good biocompatibility have been designed and utilized for targeted delivery in CVD treatments, including liposomes, polymeric nanocarriers, inorganic materials, extracellular vesicles and biomimetic membrane materials [45, 46]. These materials must meet two key criteria: low toxicity and low immunogenicity. Additionally, they should degrade into harmless metabolites *in vivo* to minimize risks from long-term accumulation [47–49]. This is particularly critical for CVD treatment, as most CVDs require long-term or even lifelong therapy. Moreover, the target sites of CVD therapy—such as atherosclerotic plaques, thrombi, ischemic myocardium or specific vascular segments—demand high local drug concentrations while requiring stringent biosafety. Therefore, biocompatibility of nanocarriers becomes even more important. Different nanocarriers exhibit distinct advantages and limitations in CVD applications. Liposomes, for example, offer excellent biocompatibility and high drug-loading capacity. However, they face challenges including short *in vivo* circulation half-life, high susceptibility to clearance by the reticuloendothelial system and targeting efficiency limited by surface modification strategies [50, 51]. Polymeric nanocarriers have unique merits: structural modifications allow precise tuning of degradation rates and targeting ligand density, along with a broad drug-loading spectrum. Nevertheless, some synthetic polymers carry potential cytotoxicity, and their long-term biosafety requires further validation [52, 53]. Inorganic nanomaterials (e.g. silica, MXene) possess good stability and unique optical/conductive properties. These features make them suitable for loading bioactive molecules or constructing smart responsive systems. Yet, their poor biodegradability raises concerns about long-term *in vivo* accumulation [54, 55]. Extracellular vesicles (particularly exosomes) show inherent cell-targeting ability and low immunogenicity, representing the highest level of biocompatibility among these carriers. However, their low yield and high isolation and purification costs partially hinder clinical translation. Biomimetic membrane materials can effectively evade immune clearance, while enhancing biocompatibility and targeting specificity. Despite these advantages, their complex preparation processes and difficulties in scalable production remain major obstacles [54].

**Figure 2:**
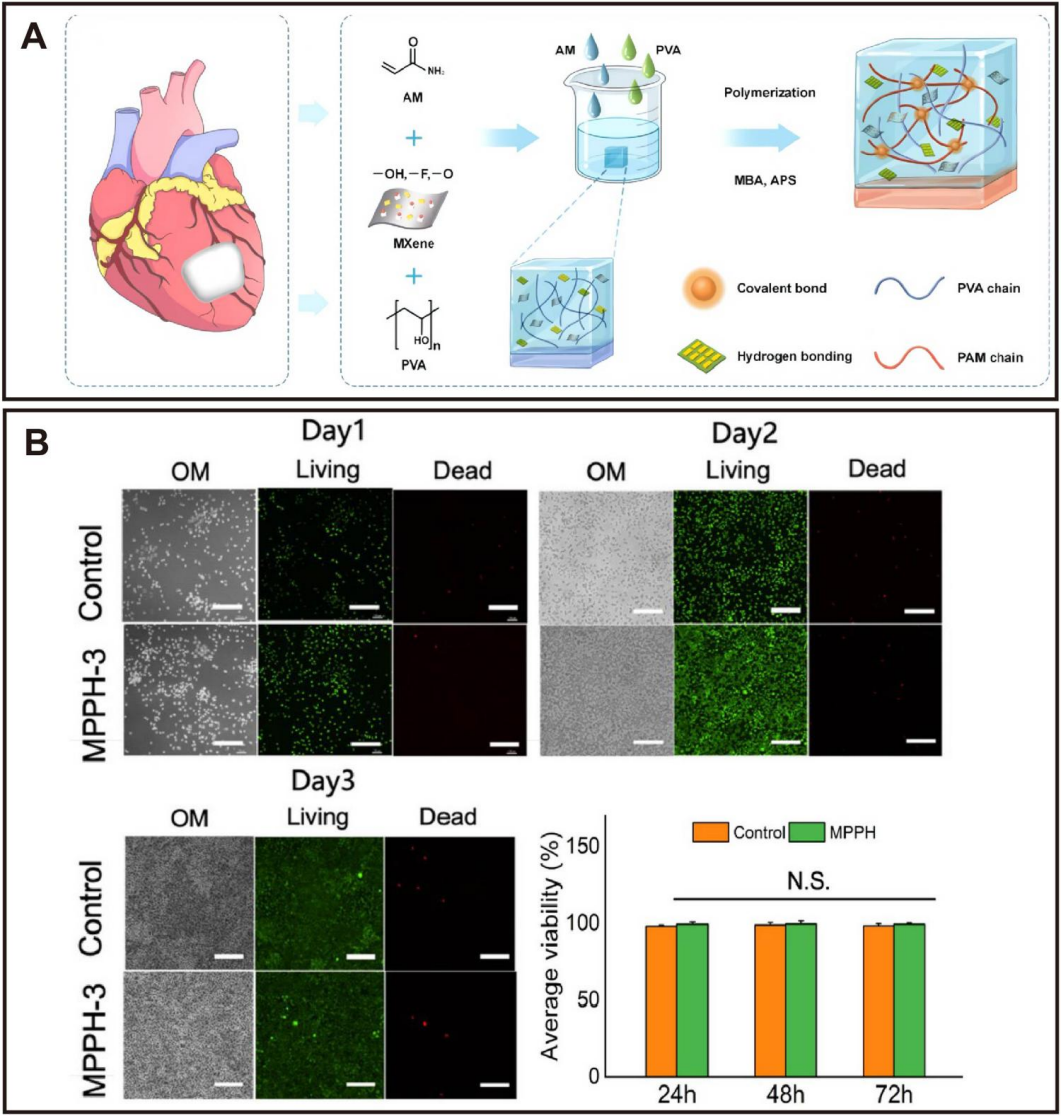
Schematic diagram of the fabrication and biocompatibility of the MXene-composite hydrogel (MPPH) cardiac patch. (**A**) Schematic of MPPH synthesis and crosslinking mechanism. (**B**) *In vitro* biocompatibility of the MPPH-3 patch. Reproduced with permission [61]. Copyright 2025, Elsevier.

While the aforementioned drug delivery systems are each suited for systemic administration or localized precision delivery, they may not fully address the needs of conditions like myocardial infarction, which requires a material capable of providing both mechanical support and tissue repair. Hydrogels, with their high-water content, can mimic the natural extracellular matrix of cardiac tissue while providing mechanical support, making them ideal candidate carriers for myocardial infarction repair therapy [56, 57]. Research shows that hydrogels implanted *in vivo* exhibit excellent biocompatibility and tunable degradation properties, capable of promoting tissue regeneration and reducing immune reactions by loading healthy functional cells [58]. Recent optimizations in cardiac tissue engineering have led to the development of hydrogel-based cardiac patches that balance biocompatibility, conductivity and mechanical properties. Examples include chitosan-selenium nanoparticle films, 3D-printed conductive hydrogels and MXene-composite hydrogel cardiac patches [59–61] (Figure 2).

### Precise control of size and morphology

The size distribution of nanocarriers significantly influences their *in vivo* behavior. Nanocarriers with an optimal size distribution can more easily penetrate physiological barriers and avoid recognition and capture by the mononuclear phagocyte system, thereby prolonging their circulation time in the bloodstream [62]. The size-effect relationship is not simply linear but highly dependent on the specific biological system and pathophysiological state. Studies indicate that capillary endothelial fenestrations allow extravasation of particles with hydrodynamic diameters less than 10 nm via paracellular pathways. Endothelial cells exhibit highest endocytic efficiency for particles sized 50–70 nm. Regarding clearance pathways, particles smaller than 6 nm are rapidly excreted by the kidneys, while those between 100 nm and 150 nm are primarily sequestered by the liver [63]. Under pathological conditions, such as in atherosclerotic plaques, widened endothelial gaps allow infiltration of lipoproteins around 25 nm into the subendothelial space. Consequently, recombinant high-density lipoprotein nanocarriers targeting such lesions are typically designed within the 7–30 nm range, whereas particles larger than 30 nm are often used for nontargeted delivery or as imaging probes [64, 65]. Beyond size, particle shape also significantly affects hemodynamic behavior. Nonspherical particles like rods or disks can adjust their flow orientation and increase effective contact area with the vessel wall, thereby prolonging circulation time and enhancing targeted adhesion capabilities, often outperforming traditional spherical particles [62]. Therefore, given the complex interactions between nanocarriers and the microenvironment, systematic optimization of parameters like size and morphology based on the specific pathophysiological characteristics of the disease is essential for achieving efficient delivery.

### Tissue and cell-specific targeting

**Figure 3:**
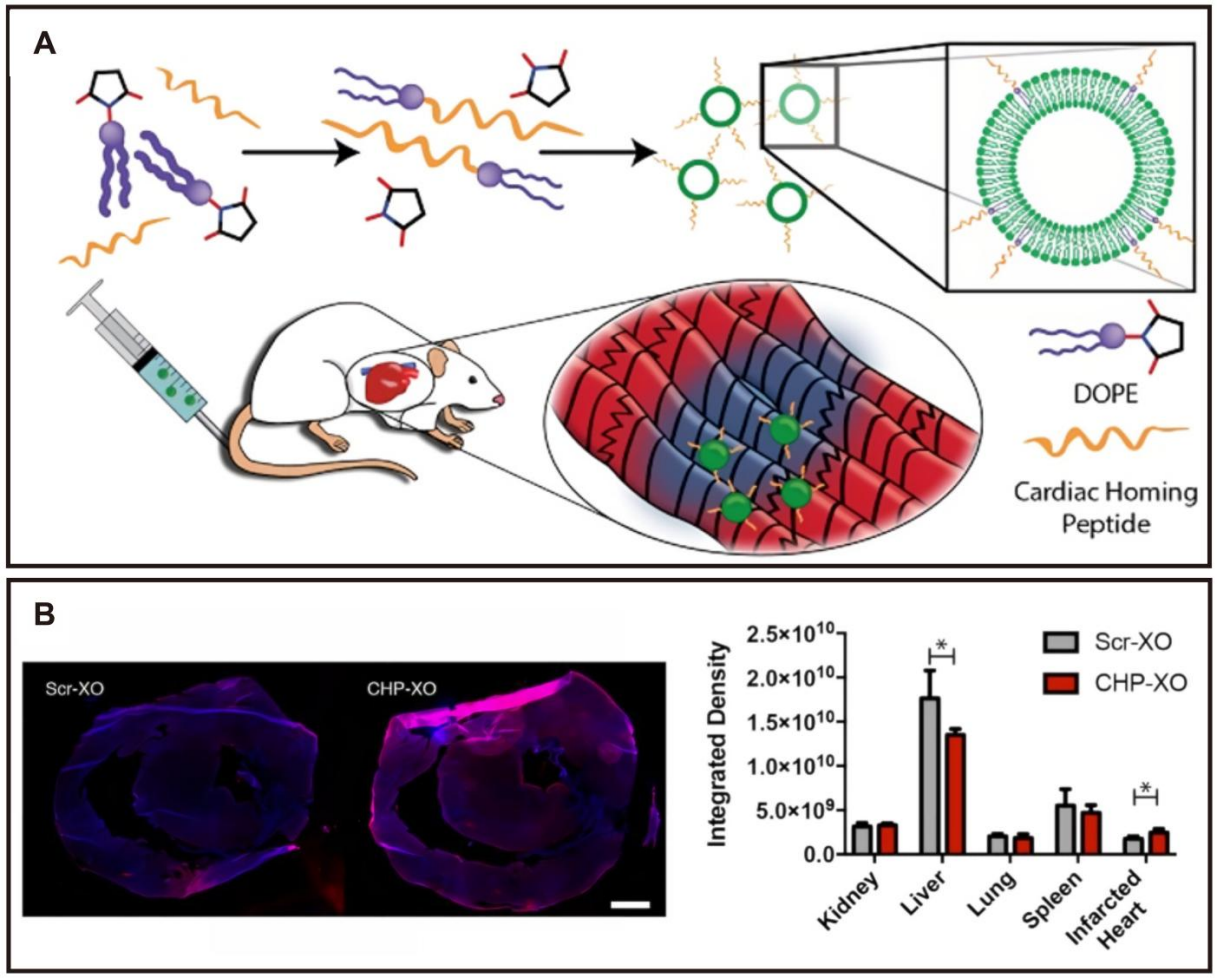
Preparation and *in vitro* targeting validation of cardiac homing peptide-modified exosomes (CHP-XO). (**A**) Schematic of CHP-targeted exosome construction. (**B**) Increased retention of CHP-tagged exosomes in *ex vivo* labeled infarcted rat heart slices [69]. Copyright 2018, Ivyspring International Publisher.

Targeting pathological features of the cardiovascular system and cell-specific surface markers can effectively enhance the selective retention of nanomedicines in lesion areas and their cellular internalization efficiency. Particularly in therapies targeting heart-specific molecules, cardiac-directed delivery strategies help reduce dosage requirements and minimize potential adverse effects on other organs [66]. Recent rapid development of cardiac targeting peptides (CTPs) and related technologies has opened new avenues for treating ischemic heart disease. CTP is a 12-amino acid unnatural peptide identified through phage display technology, capable of specifically recognizing the KCNH5 receptor on cardiomyocytes, thereby enabling precise myocardial-directed drug delivery [67, 68]. In models of ischemic cardiomyopathy and heart failure, conjugates of CTP with active therapeutic drugs significantly reduce drug distribution in nontarget organs, improving therapeutic efficacy and reducing systemic toxicity [66, 69, 70] (Figure 3). Furthermore, to address the complex pathological microenvironment of ischemic myocardium, researchers have developed various delivery strategies based on different targeting mechanisms. These include targeting specific receptors such as atrial natriuretic peptide, angiotensin II type 1 receptor, vascular cell adhesion molecule-1 and P-selectin, as well as smart delivery carriers responsive to factors in the pathological microenvironment like pH, ROS and shear stress [13, 71–77]. The establishment of these diverse cardiac targeting strategies provides new insights for advancing precision medicine in heart disease.

### Selection of mitochondria-targeting ligands

The selection of mitochondria-targeting ligands refers to choosing ligands (typically small molecules, peptides or other compounds) that can effectively enter mitochondria to achieve specific therapeutic effects or modulate mitochondrial function. These ligands usually possess special structures or functions enabling them to cross cell and mitochondrial membranes and ultimately function within mitochondria. Here, we summarize common types of mitochondria-targeting ligands, their mechanisms of action and application examples.

#### TPP^+^-based ligands

The triphenylphosphonium cation (TPP^+^) is one of the most widely used small molecule ligands in mitochondria-targeted nanocarriers. As a highly lipophilic cation, TPP^+^ can passively diffuse across cell membranes and accumulate in the mitochondrial matrix under the drive of mitochondrial membrane potential. This process depends on the electrochemical gradient principle rather than specific receptors or active transporters. It, thus, enables TPP^+^ and its conjugates to accumulate selectively in various cell types, with mitochondrial concentrations potentially reaching hundreds of times that of the extracellular environment [78]. By covalently linking TPP^+^ to bioactive molecules, various mitochondria-targeted drugs have been constructed, including MitoQ (TPP^+^-ubiquinone conjugate), Mito-TEMPOL (TPP^+^-nitroxide), SkQ1 (TPP^+^-benzothiophene derivative) and Mito-ChM (TPP^+^-vitamin E analog) [79–82]. Additionally, the mitochondrial accumulation property of TPP^+^ is also utilized in designing highly sensitive probes for diagnosis and imaging [83].

Among the developed TPP^+^-modified mitochondria-targeting compounds, MitoQ is the most systematically studied candidate in clinical translation, with trials primarily focusing on vascular aging and chronic degenerative CVDs (Table 1). This focus on chronic conditions is closely tied to the mechanism of TPP^+^ accumulation. During acute ischemia, the rapid loss of mitochondrial membrane potential drastically reduces TPP^+^ uptake. In contrast, the mitochondrial membrane potential is often partially preserved during chronic disease progression, which generally supports sufficient accumulation of TPP^+^ and similar lipophilic cations [84]. However, the mitochondria-targeted antioxidant effects of MitoQ are mainly observed in models such as age-related endothelial dysfunction and exercise-induced DNA damage. Its efficacy is limited, though, in disease states with more complex pathological mechanisms or in those where ROS is predominantly derived from nonmitochondrial sources [85, 86]. Based on the current scope of evidence and its limitations, future research on complex diseases involving multiple overlapping mechanisms could consider combination therapeutic strategies. For instance, MitoQ can be used to scavenge mtROS and alleviate oxidative damage. It can be combined with metformin to activate the AMPK signaling pathway, thereby promoting mitochondrial biogenesis and autophagy. Alternatively, MitoQ can be paired with NADPH oxidase inhibitors to synergistically suppress nonmitochondrial ROS production [87, 88].

**Table 1:** Clinical trials reported on ClinicalTrials.gov for TPP^+^-conjugated compounds in the treatment of CVDs.

	Trial title	Condition	Compound	Status	Primary outcome
1	Impacts of Mitochondrial-targeted Antioxidant on Peripheral Artery Disease Patients (NCT03506633)	Peripheral artery disease	MitoQ	Completed	Endothelial function (flow-mediated dilation): MitoQ, 6.13 ± 2.1; Placebo, 3.78 ± 1.13 (between-group statistics not reported)
2	MitoQ and Exercise Effects on Vascular Health (MITO-STEP, NCT05686967)	Endothelial function in postmenopausal women	MitoQ	Active, not recruiting	–
3	Examining the Effects of Mitochondrial Oxidative Stress in DCM (MitoDCM, NCT05410873)	Dilated cardiomyopathy	MitoQ	Completed	Not provided
4	The Efficacy of Oral Mitoquinone Supplementation for Improving Physiological in Middle-aged and Older Adults (NCT02597023)	Aging	MitoQ	Completed	MitoQ improved flow-mediated dilation by 42% vs. placebo at 6 weeks (*P *< 0.05).
5	Role of Mitochondrial-derived Oxidative Stress to Promote Vascular Endothelial Dysfunction in Nonexercisers With Aging (NCT05872139)	Aging, arterial stiffness, endothelial dysfuunction	MitoQ	Completed	Not provided
6	Mitochondrial-targeted Antioxidant Supplementation for Improving Age-related Vascular Dysfunction in Humans (NCT04851288)	Aging	MitoQ	Recruiting	–
7	The Mito-Frail Trial: Effects of MitoQ on Vasodilation, Mobility and Cognitive Performance in Frail Older Adults (NCT06027554)	Aging	MitoQ	Recruiting	–
8	MitoQ Treatment of Claudication: Myofiber and Micro-vessel Pathology (NCT06409949)	Peripheral Artery Disease	MitoQ	Recruiting	–

#### Heterocyclic aromatic cations

Heterocyclic aromatic cations are also lipophilic and can accumulate in the mitochondrial matrix leveraging the high membrane potential. They are commonly used for mitochondrial labeling and drug delivery. Reported heterocyclic cations for mitochondrial targeting include rhodamine derivatives, cyanine derivatives, dequalinium, cationic indole derivatives and pyridinium cations, among others [78]. By introducing different functional groups, these cations can be flexibly designed for specific mitochondrial targeting and functional intervention. For example, introducing a butyl ester group of rhodamines 19 enables binding to mitochondrial ATP synthase and induces uncoupling [89]; constructing metal complex systems can convert rhodamine derivatives into mitochondria-targeted photodynamic therapeutic agents [90].

#### Mitochondrial targeting sequence

**Figure 4:**
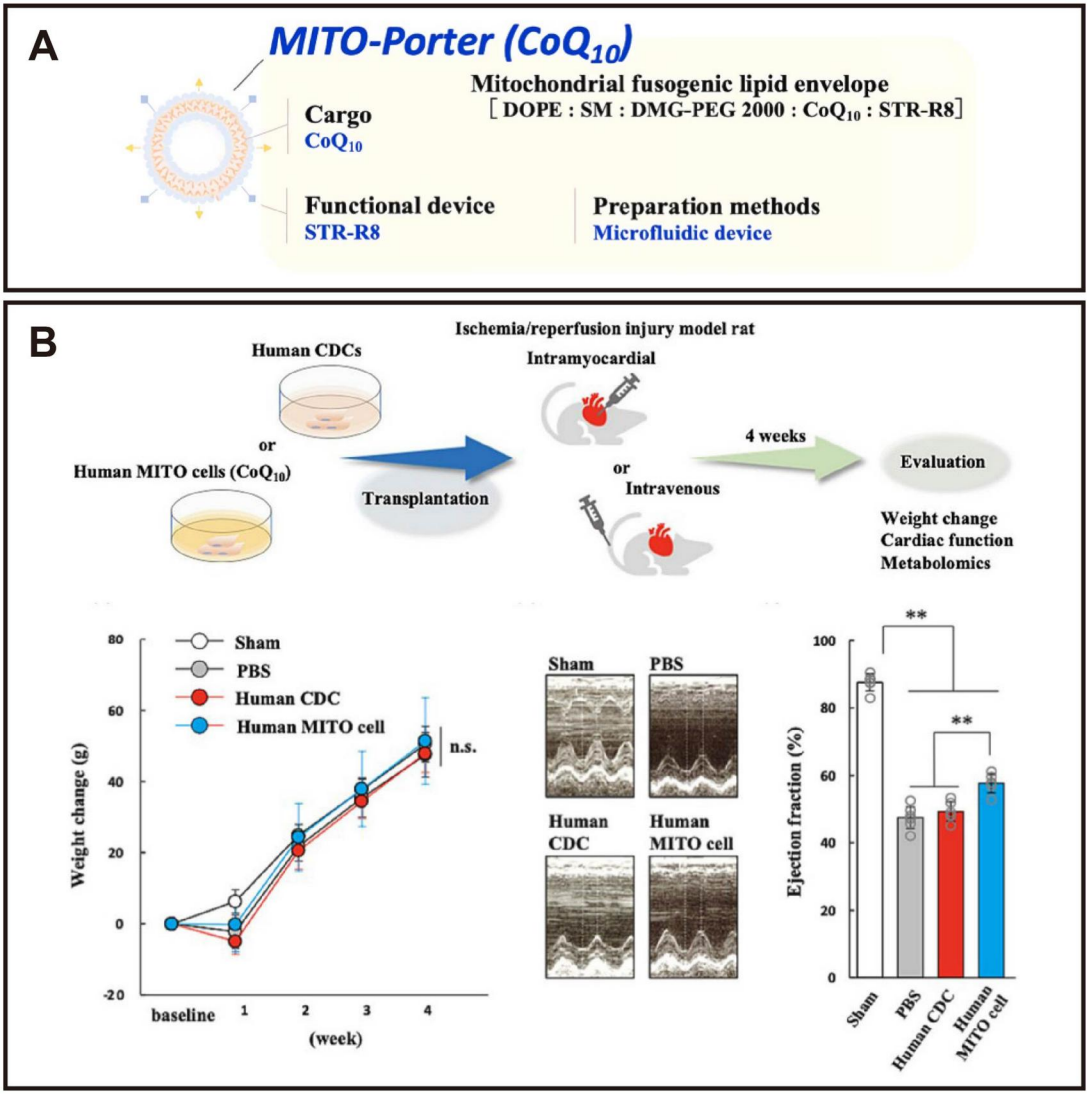
Schematic diagram of the study using MITO-Porter to activate mitochondria in human cardiosphere-derived cells for myocardial regeneration therapy. (**A**) Construction of MITO-Porter (CoQ10). (**B**) Assessment of therapeutic efficacy of human MITO cells (CoQ10) by intramyocardial injection in a rat model of myocardial ischemia-reperfusion. Reproduced with permission [14]. Copyright 2024, Elsevier.

The mitochondrial targeting sequence (MTS) is a short peptide, rich in basic and hydroxylated amino acids, located at the N-terminus of nuclear-encoded mitochondrial proteins. It primarily mediates the translocation of these proteins into mitochondria [44]. This sequence can spontaneously form an amphipathic α-helix, where the positively charged side responds to the mitochondrial membrane potential and the hydrophobic side facilitates interaction with the lipid environment of mitochondrial membranes, collectively promoting protein translocation across the membranes. Once the protein enters the mitochondrial matrix, its N-terminal MTS is specifically cleaved by matrix peptidases, preventing the protein from returning to the cytoplasm [44, 91]. Based on the membrane potential-dependent targeting mechanism of MTS, researchers have designed highly positively charged peptides (e.g. octa-arginine) mimicking its function to construct mitochondria-targeted drug carriers, such as the MITO-Porter system [14] (Figure 4). A study by Hibino et al. demonstrated that encapsulating and delivering CoQ10 via MITO-Porter enables more effective drug transport to mitochondria in macrophages, compared to the administration of free CoQ10 alone. This strategy significantly enhanced mitochondrial respiration and exerted marked antioxidant effects [92]. Although this strategy shows promise, it faces limitations. The MTS itself has limited ability to cross the plasma membrane and often requires combination with cell-penetrating peptides to enhance delivery efficiency. Moreover, studies show that not all selected MTS sequences possess effective mitochondrial targeting capability, necessitating thorough validation during screening and design [93].

#### Mitochondria-targeting peptides

Mitochondria-targeting peptides mainly include naturally occurring Mitochondrial-Derived Peptides (MDPs) and artificially designed synthetic peptides. Natural MDPs are encoded by small open reading frames within mtDNA and translated by mitochondrial ribosomes, playing key roles in cellular stress response and mitochondria-nucleus retrograde signaling [94]. Three major human MDPs identified to date are: (i) Humanin (HN), encoded from the mtDNA 16S rRNA region, existing as secreted peptides of 21 or 24 amino acids. HN exerts anti-apoptotic and cytoprotective functions by forming receptor complexes with intracellular molecules and cell surface ligands, showing neuroprotective and cytoprotective activities in models of Alzheimer’s disease, ischemia-reperfusion injury and diabetes [95–98]; (ii) Humanin-like peptides (SHLPs 1-6), also encoded from the mtDNA 16S rRNA region. SHLP2 and SHLP3 exhibit protective effects similar to HN. They significantly inhibit apoptosis and ROS generation, while improving mitochondrial metabolism and insulin sensitivity [99]; (iii) MOTS-c, a 16-amino acid peptide encoded by the mtDNA 12S rRNA gene. Under stress or exercise conditions, MOTS-c can translocate to the nucleus, directly regulating nuclear gene expression. It modulates energy metabolism via the Folate-AICAR-AMPK pathway, promoting glucose uptake and enhancing insulin sensitivity. It also suppresses excessive inflammation and inhibits aging-related pathological processes, thus, showing potential therapeutic value for obesity, diabetes and sarcopenia [100–102].

Chemically synthesized Mitochondria-Targeting Peptides (MTPs) are often designed based on Cell-Penetrating Peptide (CPP) structures for delivering exogenous functional molecules. Unlike typical CPPs that rely on membrane potential, MTPs often achieve mitochondrial specificity by incorporating MTS motifs or optimizing charge distribution. Their entry process is less dependent on the mitochondrial membrane potential, retaining delivery potential under pathological conditions [78, 93, 103]. The Szeto-Schiller (SS) peptide family is a classic example of MTPs, with SS-31 (also known as elamipretide, Bendavia™ or MTP-131) being the most extensively studied. SS-31 acts by specifically binding to cardiolipin, exerting dual effects: it prevents cardiolipin-mediated cytochrome c peroxidase activation, inhibiting apoptosis; and it stabilizes respiratory chain complexes located on the cristae, enhancing OXPHOS efficiency and suppressing excessive ROS production. Another representative synthetic molecule, KH176, primarily targets mitochondrial redox regulation for treating inherited mitochondrial diseases caused by mtDNA mutations [104, 105]. Its mechanism involves promoting thioredoxin regeneration and maintaining NADPH in its reduced state, thereby enhancing oxidative stress tolerance [106]. Its active metabolite KH176m was also found to selectively inhibit microsomal prostaglandin E synthase-1, blocking prostaglandin E2 biosynthesis, suggesting potential therapeutic value in inflammation-related diseases [107]. SS-31 has shown therapeutic potential in various disease models, including heart failure, kidney injury and atherosclerosis, and has entered multiple Phase II/III clinical trials [108–113]. However, it failed to meet primary endpoints in trials for primary mitochondrial myopathy (MMPOWER-3) and heart failure with reduced ejection fraction (PROGRESS-HF) [112, 113] (Table 2).

**Table 2:** Clinical trials reported on ClinicalTrials.gov for mitochondria-targeting peptides in the treatment of CVDs.

	Trial title	Condition	Compound	Status	Primary outcome
1	Evaluation of Myocardial Effects of MTP-131 for Reducing Reperfusion Injury in Patients with Acute Coronary Events (NCT01572909)	Reperfusion Injury, STEMI	MTP-131	Completed	Infarct size (CK-MB AUC 72 h): MTP-131, 5570 ng·h/mL vs. placebo, 5785 ng·h/mL (*P* = NS)
2	The Impact of Intravenous Bendavia™ on Endothelial Reactivity Dysfunction in Cigarette Smoking (NCT01518985)	Endothelial reactivity dysfunction, Smoker	MTP-131	Completed	Not provided
3	Effect of Elamipretide on Left Ventricular Function in Subjects with Stable Heart Failure with Reduced Ejection Fraction (The PROGRESS-HF Phase 2 Trial, NCT02788747)	Heart Failure	MTP-131	Completed	No significant improvement in left ventricular end-systolic volume was observed with MTP-131 compared to placebo.
4	A Study to Evaluate the Effects of 4 Weeks Treatment with Subcutaneous Elamipretide on Left Ventricular Function in Subjects with Stable Heart Failure with Preserved Ejection Fraction (NCT02814097)	Chronic Heart Failure	MTP-131	Completed	Not provided
5	A Phase 2 Study to Evaluate the Cardiac and Renal Effects of Short-Term Treatment with Elamipretide in Patients Hospitalized with Congestion Due to Heart Failure (NCT02914665)	Chronic Heart Failure	MTP-131	Completed	Not provided
6	A Study Investigating the Safety, Tolerability and Pharmacokinetics of MTP-131 in Subjects with Congestive Heart Failure (NCT02388464)	Congestive Heart Failure	MTP-131	Completed	At the end of a 4-h infusion, the highest dose of MTP-131 (0.25 mg·kg^−1^·h^−1^) produced significant, transient and dose-dependent reductions in left ventricular volumes compared to placebo.
7	A Trial to Evaluate Safety and Efficacy of Elamipretide Primary Mitochondrial Myopathy Followed by Open-Label Extension (MMPOWER3, NCT03323749)	Primary Mitochondrial Myopathy	MTP-131	Terminated	Co-primary endpoints: 6-min walk test change (*P *= 0.69) and PMSA fatigue score change (*P *= 0.81) were both nonsignificant vs. placebo.
8	The KHENERGYZE Study (NCT04165239)	Mitochondrial Myopathy	KH176	Completed	Not provided

The negative results of these two trials highlight the challenges in translating positive preclinical signals into definitive clinical benefits and prompt profound reflections on key aspects of drug development, including dosage selection, patient population, treatment timing and endpoint setting. From the perspective of dosage and treatment duration, the effective doses identified in preclinical studies are difficult to extrapolate directly to humans. There are fundamental differences between rodents and humans in the basic metabolic characteristics of cardiac mitochondria, the nature of the pathophysiological processes in the modeled diseases, and the composition and response patterns of the immune system. These biological interspecies disparities may directly undermine the translational relevance of the drug doses and efficacy signals established in preclinical research [114]. In the PROGRESS-HF trial, both 4 mg/day and 40 mg/day subcutaneous injection regimens failed to significantly improve left ventricular end-systolic volume; the low dose may have failed to reach the effective threshold due to insufficient bioavailability, while the high dose only increased injection site adverse reactions without establishing a reasonable dose-effect curve [112]. Additionally, preclinical studies have shown that SS-31 typically requires continuous administration for several months to exert cardioprotective effects [115], whereas the 4-week treatment duration in PROGRESS-HF was likely insufficient to reverse established chronic cardiac remodeling. Regarding treatment timing and endpoint setting, the core mechanism of SS-31 lies in inhibiting mitochondrial apoptosis and reducing oxidative stress, making it more suitable for early intervention in acute injuries. However, PROGRESS-HF focused on stable heart failure, where the core pathological changes have shifted to chronic remodeling, and mitochondrial dysfunction is mostly a secondary manifestation. Meanwhile, the trial relied on traditional cardiac function indicators and did not incorporate specific biomarkers of mitochondrial function, which may have prevented the accurate capture of the drug’s mechanistic benefits [112]. Genetic heterogeneity of the patient population is another crucial lesson. The MMPOWER-3 trial adopted a “basket design” enrolling patients with distinctly different pathological mechanisms: 72.9% with mtDNA mutations and 27.1% with nuclear DNA (nDNA) mutations [113]. SS-31 exerts its effects by binding to cardiolipin. This mechanism may be more compatible with patients with nDNA mutations (especially those affecting mtDNA maintenance). However, it has limited efficacy in repairing damage caused by mtDNA mutations, leading to the dilution of overall therapeutic signals. Notably, post-hoc analysis of the trial provided evidence for this mechanistic specificity. When the nDNA subgroup was precisely stratified to patients with defects in mtDNA polymerase-related genes (e.g. POLG, TWNK), the improvement in 6-min walk test results approached statistical significance. Additionally, pharmacokinetic analysis observed a weak correlation trend between drug exposure and efficacy only in the nDNA subgroup [116]. These data consistently demonstrate that the drug’s therapeutic signals are highly concentrated in specific patient subgroups with mtDNA maintenance disorders caused by nuclear gene defects. Therefore, future research should shift toward a mechanism-oriented precision development strategy, involving patient stratification based on the genetic background and pathological characteristics of the disease, optimization of dosage regimens and treatment durations, and prioritization of clinical trials in mechanistically compatible subtypes.

#### Mitochondria-targeting peptoids

Due to the susceptibility of cationic peptides to proteolytic hydrolysis, researchers have developed mitochondria-targeting peptoids with anti-protease hydrolysis properties [117, 118]. These peptoids feature a backbone of oligo-N-substituted glycines with side chains attached to the backbone nitrogen atoms. The lack of Cα chiral centers and backbone N-H hydrogen bond donors confers high stability against protease degradation. Their structures can be further customized into amphipathic conformations. This structural design enables efficient biomembrane penetration and mitochondrial-directed delivery, with the added benefit of low cytotoxicity [118]. Structure-activity relationship studies indicate that the targeting efficiency of these peptoids is closely related to chain length and spatial conformation. Among various structures, dodecamers are identified as the optimal choice, balancing helical folding and membrane penetration ability. Regarding safety, this carrier causes only mild perturbation of the mitochondrial membrane potential (ΔΨm retention >84%), does not affect mitochondrial respiratory function, and shows no significant cytotoxicity. Compared to the easy degradation of traditional cationic peptides and potential toxicity issues with TPP^+^ conjugates, these peptoids, with their stable helical conformation and enzyme resistance, offer a promising new strategy with translational potential for mitochondria-targeted nanocarriers [118].

### Loaded active therapeutic agents

Upon successful targeting of nanomedicines to mitochondria, the loaded active therapeutic components become the core executors for reversing mitochondrial dysfunction, restoring cardiac energy metabolism and regulating cell fate. These therapeutic agents are diverse, with varied mechanisms of action, precisely intervening in different aspects of mitochondrial pathophysiology. Small molecule drugs are currently the most widely used type. Among them, antioxidants (e.g. metformin, CoQ10, diosgenin, resveratrol) can directly neutralize excess ROS within mitochondria, alleviating oxidative stress damage [13, 14, 119–122]. Meanwhile, mitochondrial permeability transition pore (mPTP) inhibitors (e.g. cyclosporine A) inhibit aberrant mPTP opening, preventing cytochrome c release and apoptosis pathway activation, thereby maintaining mitochondrial membrane potential stability and cardiomyocyte survival [123, 124]. Macromolecular therapeutics, including functional proteins and nucleic acid drugs, offer the potential for deeper intervention in mitochondrial homeostasis. They can precisely interfere with gene expression and signal transduction, correcting pathological processes at their source [25, 72, 125–127]. Synthetic bioactive materials represent an emerging strategy, possessing designed biological functions themselves. For instance, various nanozymes can mimic the catalytic activity of natural antioxidant enzymes, continuously scavenging ROS; self-assembling nanomaterials can integrate small molecule antioxidants with microenvironment-responsive units, achieving efficient ROS clearance and controlled drug release at target sites [128–130]. Furthermore, therapies based on natural active ingredients also demonstrate multidimensional repair potential. Mesenchymal stem cells exert multidimensional repair effects via dual pathways. They can directly provide functional mitochondria to damaged myocardium through mitochondrial transplantation, which supplements energy and inhibits oxidative damage [131]. Meanwhile, their derived exosomes serve as natural delivery vehicles: by transferring proteins, miRNAs and other active substances, these exosomes indirectly regulate mitochondrial function, improve the cardiac microenvironment and synergistically promote tissue repair [132–135] (Table 3).

**Table 3:** Laboratory-stage nano-loaded active therapeutic agents targeting mitochondrial dysfunction.

Category	Active therapeutic agent	Mechanism of mitochondrial targeting action	Model	References
Metformin and Melatonin	Metformin	1. Reduces mtROS production, protects ΔΨm. 2. Induces protective autophagy via the AMPK-mTOR-ULK1 pathway.	Dox-induced cardiotoxicity model in mice	[122]
	Metformin and Melatonin	1. Regulates autophagy via AMPK pathway activation, maintaining mitochondrial homeostasis. 2. Scavenges ROS to alleviate oxidative stress.	Mouse MI model	[121]
	CoQ10	1. Scavenges mtROS. 2. Enhances mitochondrial basal respiration, OXPHOS-dependent ATP production and maximal respiratory capacity.	Mouse primary bone marrow-derived macrophages	[14]
	CoQ10 and SS31	1. Scavenges mtROS. 2. Inhibits mPTP opening, restores ΔΨm, mitigates mitochondrial oxidative damage and reduces apoptosis.	Mouse heterotopic heart transplantation cold ischemia-reperfusioninjury model	[120]
	Cyclosporine A	1. Binds cyclophilin D in the mitochondrial matrix to inhibit mPTP opening, maintaining ΔΨm. 2. Reduces mtROS generation and inhibits cardiomyocyte apoptosis.	Rat I/R model	[123, 124]
	Mdivi1	1. Targets and inhibits the mitochondrial fission protein Drp1. 2. Blocks Bax recruitment to mitochondria and its oligomerization, inhibiting mitochondrial outer membrane permeabilization. 3. Reduces cytochrome c release, inhibits caspase-dependent apoptosis and maintains mitochondrial structural integrity.	Mouse myocardial I/R model	[136]
	C-176 (STING inhibitor	Inhibits the mtDNA-mediated inflammatory pathway, alleviating mitochondrial dysfunction-associated myocardial injury.	Mouse model of experimental autoimmune myocarditis	[127]
	Dioscin	Reduce the production of mitochondrial superoxides, restore mitochondrial membrane potential and repair damaged mitochondrial function	Mouse MI model	[13]
	Resveratrol	Enhance the maximum respiratory capacity and reserve respiratory volume of mitochondria	Rat H9c2 cells	[119]
	Berberine	1. Regulate the polarization of macrophages from the M1 to the M2 type, reduce the release of pro-inflammatory factors and increase the secretion of anti-inflammatory factor. 2. Alleviate mitochondrial dysfunction mediated by inflammation and inhibit cardiomyocyte apoptosis. 3. Indirectly improve the mitochondrial-related microenvironment for myocardial repair.	Rat model of MI	[137]
	Curcumin	Inhibit the degradation of GPX4 involved in ferroptosis and maintain the integrity of the mitochondrial membrane	LPS-induced myocardial injury model of sepsis in mice	[138]
	Curcumin	1. Remove mtROS and NO, reduce lipid peroxidation and protect mitochondrial membrane integrity. 2. Increase the level of reduced glutathione and enhance the mitochondrial antioxidant defense. 3. Restore the activity of mitochondrial Na^+^/K^+^-ATPase and improve the mitochondrial energy metabolism function.	Dox-induced rat cardiotoxicity model	[139]
Biological macromolecules	Fibroblast growth factor 21	1. Up-regulate genes related to mitochondrial energy metabolism, enhancing the energy synthesis function of mitochondria. 2. Reduce ROS damage, protect the integrity of mitochondrial membrane structure and prevent mitochondrial swelling and rupture. 3. Improve the normal arrangement of mitochondria and myocardial fibers and restore the physiological morphology of mitochondria.	Mouse myocardial I/R injury model	[127]
	S1pr2-siRNA	1. Inhibit the RHO/ROCK1/DRP1 signaling pathway 2. Reduce excessive mitochondrial fission, protect mitochondrial membrane integrity and inhibit mtDNA release. 3. Block the activation of NLRP3 inflammasome and pyroptosis and improve mitochondrial dysfunction-related myocardial injury.	Mouse myocardial I/R injury model	[126]
	Cysteine -γ -lyase plasmid	1. Promote the generation of endogenous H2S and protect the ultrastructure of mitochondria. 2. Inhibit excessive mitochondrial autophagy. 3. Restore the ATP synthesis ability of mitochondria. 4. By inhibiting the CHOP/GRP78/elF2α signaling pathway, regulate endoplasmic reticulum stress and mitochondrial function and reduce cardiomyocyte apoptosis.	Mouse myocardial I/R injury model	[72]
	miR-10a	1. Up-regulate mitochondrial biogenesis genes and genes related to FAO/OXPHOS, to restore mitochondrial respiratory function. 2. Increase the supply of acetyl coenzyme A. 3. Restore the expression of mitochondrial transcription factor TFAM, protect mitochondrial function and reduce the production of ROS.	ApoE−/− mouse atherosclerosis model	[125]
	Gene editing tool MitoCas9	1. Specifically eliminate the m.15059G>A mutation of mtDNA copies on the MT-CYB gene and restore the function of the mitochondrial respiratory chain complex III. 2. Restore the ΔΨm, reduce the generation of mtROS and lipid peroxidation. 3. Decrease proton leakage and nonmitochondrial oxygen consumption rate and improve the efficiency of mitochondrial bioenergetics.	Monocytoid hybridoma cells carrying m.15059G>A mutant mtDNA	[25]
Synthetic bioactive materials	TPCD material	1. Efficiently eliminate excessive mtROS in and inhibit lipid peroxidation. 2. Protect the structural and functional integrity of mitochondria and alleviate myocardial damage mediated by oxidative stress.	Dox-induced mouse cardiomyopathy model	[129]
	Nanoenzyme	1. Simulate the activity of antioxidant enzymes to eliminate excessive superoxide anions from mitochondria. 2. Protect the integrity of mtDNA. 3. Maintain the function of the mitochondrial respiratory chain.	Mouse myocardial I/R injury model	[128]
	Nanoenzymes and synthetic iron chelating agents	1. Analog antioxidant enzyme activity, remove the mitochondria source excess super oxygen anion 2. Inhibition of Fenton reaction, inhibiting mitochondrial related death, protecting mitochondria ultrastructure	Mouse myocardial I/R injury model	[130]
Natural active ingredients	Mitochondria derived from mesenchymal stem cells	1. Directly replace the damaged mitochondria to maintain mitochondrial membrane potential and bioenergetic activity. 2. Reduce the generation of mtROS and inhibit oxidative stress-induced cell apoptosis. 3. Activate the ERK pathway, alleviate endothelial cell senescence and improve mitochondrial function related to angiogenesis.	Mouse myocardial I/R injury model	[131]
	Exosomes derived from human umbilical vein endothelial cells	1. Carry anti-inflammatory miRNAs such as let-7 family and miR-16 to inhibit the TLR-NF-κB signaling axis. 2. Reduce the release of pro-inflammatory factors and the generation of extracellular ROS, protecting the integrity of mitochondrial structure. 3. Decrease the release of circulating free mtDNA caused by mitochondrial damage and improve mitochondrial dysfunction.	Cardiac tissue model of immune-induced dysfunction	[132]
	Exosomes derived from induced pluripotent stem cells	1. Target the PARP1/AIFM1 axis, downregulate the expression of PARP1 in the nucleus and inhibit the translocation of AIFM1 from the mitochondria to the nucleus. 2. Restore the mitochondrial membrane potential, increase ATP production and reduce the generation of mitochondrial ROS. 3. Inhibit oxidative stress and improve mitochondrial dysfunction.	TAC-induced rat Heart failure model	[133]

Abbreviations: AMPK, adenosine monophosphate-activated protein kinase; mTOR, mechanistic target of rapamycin; ULK1, Unc-51 Like autophagy activating kinase1; TAC, transverse aortic constriction; Drp1, dynamin-related protein 1; GPX4, glutathione peroxidase 4; LPS, lipopolysaccharide; S1pr2, sphingosine-1-phosphate receptor 2; RHO, Ras homolog gene family, member A; ROCK1, Rho-associated coiled-coil containing protein kinase 1; CHOP, C/EBP homologous protein; GRP78, glucose-regulated protein 78 kDa; eIF2α, eukaryotic translation initiation factor 2 subunit alpha; FAO, fatty acid oxidation; TFAM, mitochondrial transcription factor A; MT-CYB, mitochondrially encoded cytochrome B; ERK, extracellular signal-regulated kinase; TLR, toll-like receptor; NF-κB, nuclear factor kappa-light-chain-enhancer of activated B cells; PARP1, poly(ADP-ribose) polymerase 1; AIFM1, apoptosis-inducing factor mitochondria-associated 1.

## Key factors for optimizing cardiac drug delivery

Optimizing cardiac drug delivery requires systematic consideration and overcoming a series of key factors, including the complex cardiac anatomy, the impact of hemodynamic shear stress, the endothelial barrier, hindrance by the extracellular matrix (ECM), nonspecific adsorption due to surface charge, lysosomal degradation and clearance by the cardiac immune system. Coordinated regulation of these factors is crucial for enhancing delivery efficiency [16].

### Cardiac anatomy and hemodynamic shear stress

High-velocity blood flow within the heart and turbulence at coronary artery bifurcations generate significant fluid shear stress, which is unfavorable for the margination and stable retention of nanocarriers along the vessel walls. *In vivo* nanoparticle distribution studies in zebrafish visually confirm this phenomenon. Due to faster arterial flow and higher shear stress, nanoparticle accumulation in arteries is 6-fold lower than in veins. Computational fluid dynamics analysis further confirms that regions with lower shear, such as vascular bends, are more conducive to particle accumulation [140]. The negative impact of fluid shear stress is not limited to synthetic nanocarriers but also applies to shear-sensitive bioactive substances. Studies show that during donor mitochondrial transplantation, excessive shear stress can lead to loss of mitochondrial activity, suggesting oral administration might be a more promising therapeutic route [141].

### Endothelial barrier

**Figure 5:**
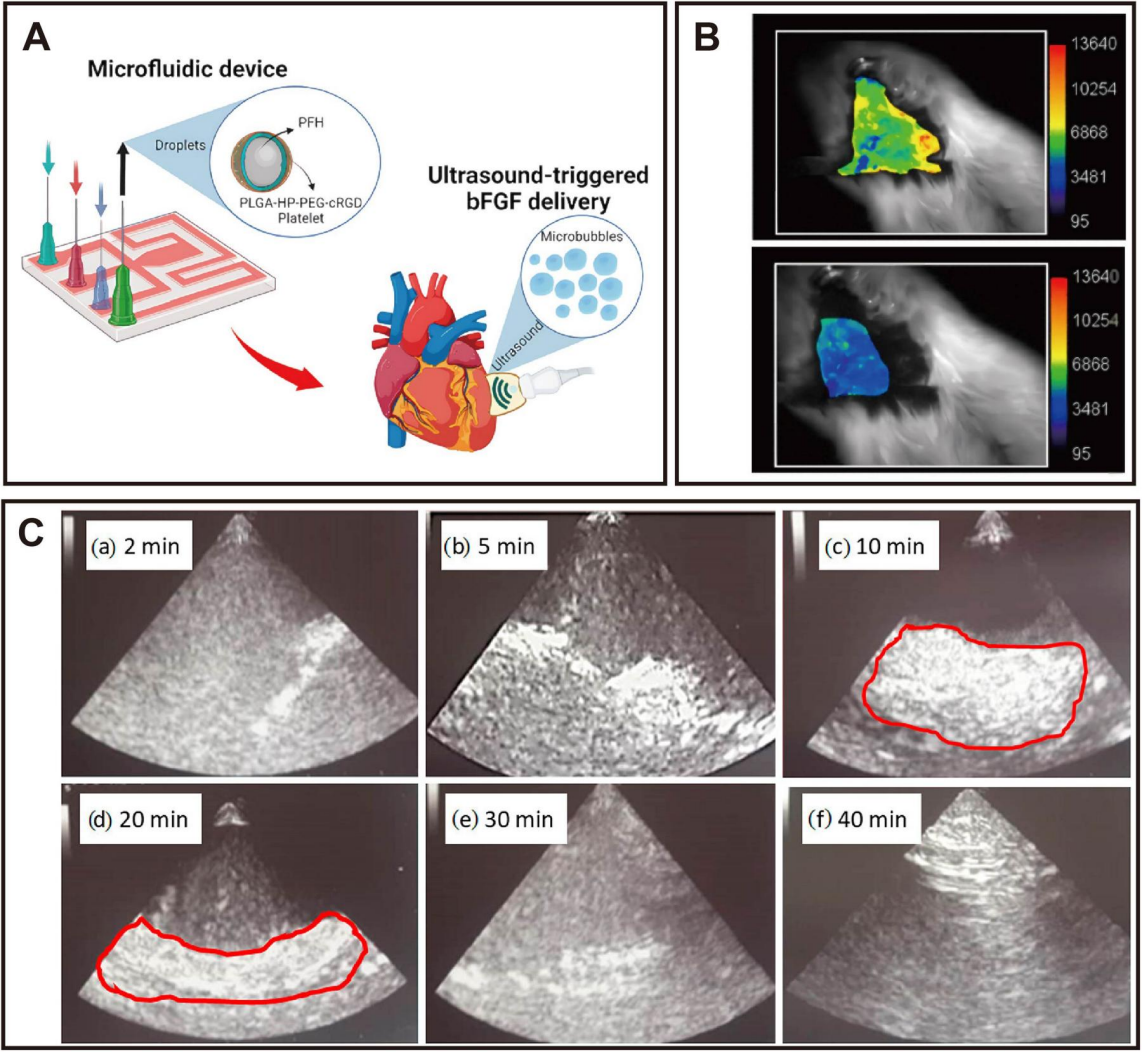
Ultrasound-triggered PFH-core-shell microbubbles for targeted bFGF delivery in myocardial infarction therapy. (**A**) Schematic illustration of the fabrication of PFH-core-shell microspheres and their ultrasound-triggered phase transition to microbubbles. (**B**) Verification of specific targeting to the ischemic myocardial region. (**C**) Ultrasound-triggered liquid-to-gas phase transition of microspheres. Reproduced with permission [143]. Copyright 2023, American Chemical Society.

Cardiac capillaries are composed of continuous endothelial cells connected by tight junctions, which strictly limit nanomedicine extravasation under steady-state conditions. Although endothelial permeability increases under pathological states like ischemia and inflammation, this opening is often heterogeneous and uncontrolled [142]. Ultrasound‑targeted microbubble destruction offers a precise physical strategy to overcome this barrier. It induces controlled microbubble expansion and rupture, which transiently and reversibly increases endothelial gaps. This further promotes localized extravasation of therapeutics. As illustrated in Figure 5, this approach is exemplified by basic fibroblast growth factor (bFGF)‑loaded, platelet‑conjugated core–shell microspheres. These particles consist of a perfluorohexane (PFH) core encapsulated in a polymeric shell that carries bFGF and surface‑anchored platelets for active targeting. Upon ultrasound exposure, the PFH core undergoes a liquid‑to‑gas phase transition, expanding the microspheres into microbubbles. Subsequent microbubble rupture not only transiently disrupts the endothelial barrier in the infarcted myocardium but also releases bFGF locally. This dual‑targeting strategy overcomes the poor specificity and off‑target effects of conventional nanocarriers, enabling precise, on‑demand drug delivery to the ischemic myocardium [143].

### Hindrance by the extracellular matrix

After successfully crossing the endothelium, nanomedicines must traverse a dense ECM network composed of collagen, fibronectin, glycosaminoglycans, etc. Its steric hindrance and electrostatic repulsion effects significantly impede nanoparticle diffusion [144]. Studies show that poly(ethylene glycol) (PEG) modification can significantly enhance the diffusion rate of nanocarriers within ECM gels. However, high diffusivity does not directly equate to high cellular uptake and the impact of ECM from different tissue sources on particle transport varies. Besides collagen, other components like glycosaminoglycans may also regulate this process [144]. Post-MI fibrosis exacerbates the ECM barrier effect, while nanocarriers coated with macrophage membranes or fused with ECM-mimetic components demonstrate better penetration capabilities [145, 146].

### Influence of surface charge

As the primary functional units of the heart, cardiomyocytes exhibit very low endocytic activity, making them inherently difficult to uptake exogenous nanocarriers, posing a challenge for drug efficacy [108]. Cellular uptake of nanocarriers is regulated not only by size and targeting specificity but also closely related to surface charge. Typically, positively charged particles can enhance uptake efficiency through electrostatic interactions with the negatively charged cell membrane [147]. However, positive charge also readily leads to serum protein adsorption forming a protein corona, affects the endocytic pathway and may cause endothelial cell dysfunction [109, 148]. Furthermore, different cell types respond differently to positively charged particles; their uptake advantage in tumor cells may not apply to the cardiovascular system [149]. To address this contradiction, charge-reversal nanocarriers have emerged. These carriers maintain a neutral or negative charge in the bloodstream for stability. Upon reaching the target microenvironment (e.g. weak acidity), they switch to a positive charge. This design cleverly balances long circulation and efficient cellular uptake [128, 150]. Regarding charge distribution, particles with anisotropic charge distribution are more likely to induce membrane deformation and endocytosis than uniformly charged structures, as the latter often lack localized interaction sites and remain trapped on the cell surface [151, 152].

### Endosomal and lysosomal escape

**Figure 6:**
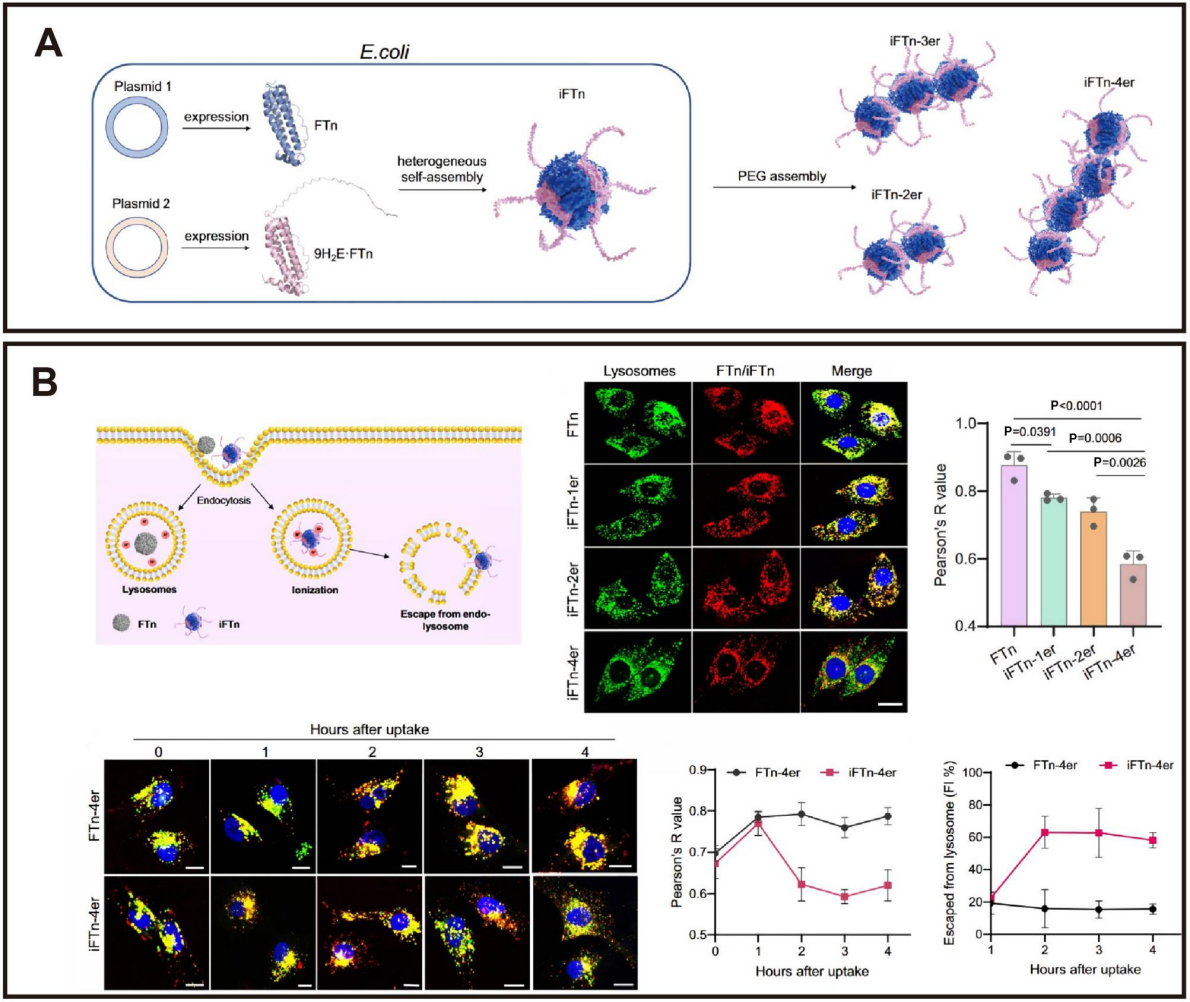
Assembly process and endosomal escape capability of iFTn and iFTn-4er. (**A**) Schematic illustration of the assembly process of iFTn and iFTn-4er. (**B**) Endo-lysosomal escape mechanism of iFTn, with verification of the specificity of lysosomal escape for the iFTn-4er variant. Reproduced with permission [128]. Copyright 2025, Springer Nature.

**Figure 7:**
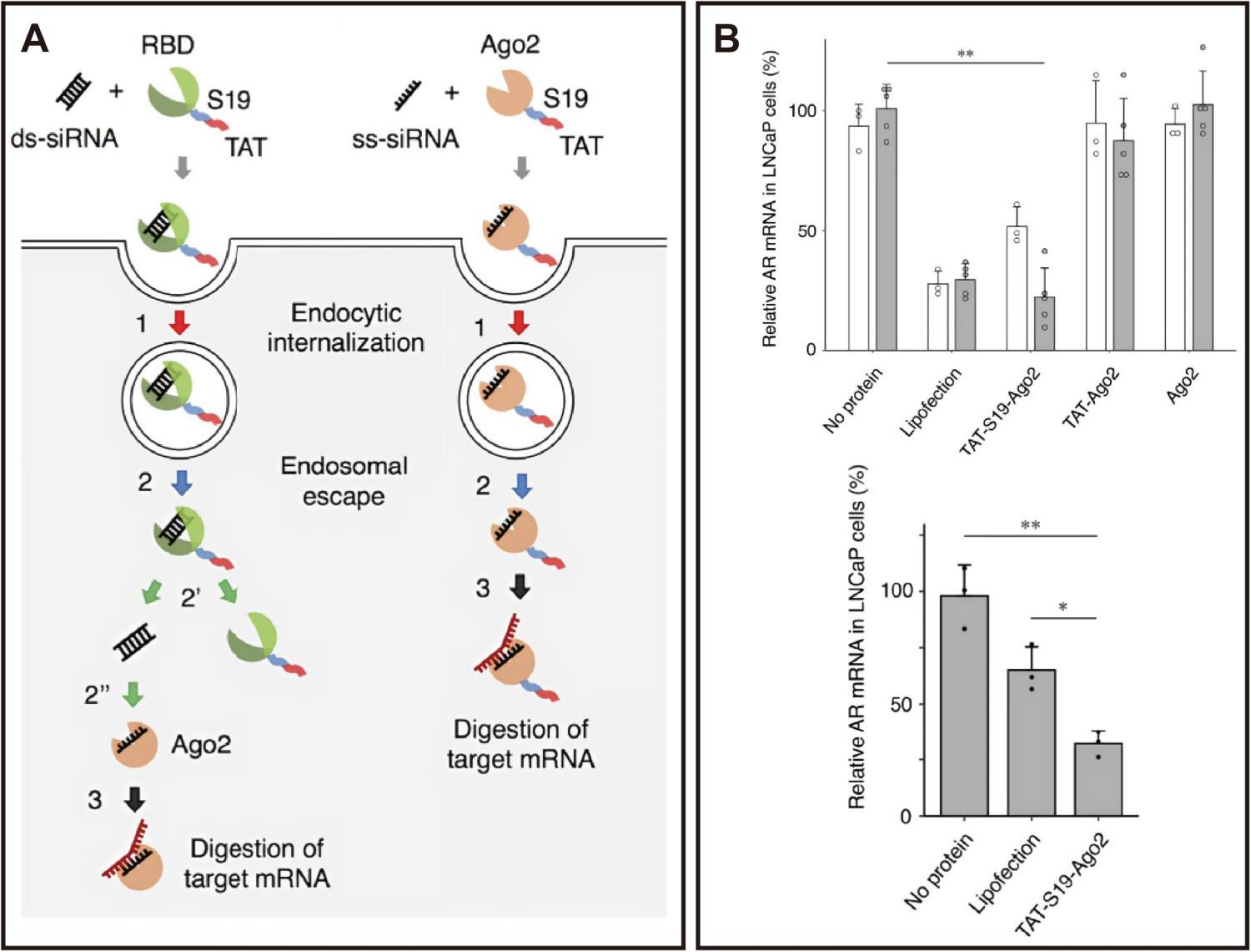
Engineered TAT-S19 fusion peptides mediate endosomal escape for efficient siRNA delivery and target gene knockdown. (**A**) Schematic illustration of the endosomal escape mechanism of TAT-S19 fusion peptides for siRNA delivery. (**B**) Quantitative analysis of AR gene knockdown efficiency in LNCaP cells. Reproduced with permission [154]. Copyright 2022, Springer Nature.

After endocytic uptake, the vast majority of nanomedicines become trapped in the endosomal-lysosomal pathway. The acidic environment and hydrolytic enzymes within lysosomes can degrade the drugs; reportedly, less than 5% of nanocarriers successfully deliver their cargo to the cytosol [153]. Therefore, efficient endo/lysosomal escape is crucial for CVD therapy. To address this bottleneck, pH-responsive membrane disruption strategies, such as engineering carriers with ionizable, histidine-rich motifs, have been developed. These motifs protonate in acidic endosomes, disrupting membrane integrity via electrostatic interactions to facilitate escape and mitigate the risks of cargo inactivation or harmful free radical generation within lysosomes. A representative example of this strategy is the work of Liu et al., [128] who genetically incorporated a histidine-rich, ionizable repeat sequence (9H2E) into human heavy-chain ferritin (FTn) to construct ionizable nanocages (iFTn). The iFTn exhibits pH-dependent charge reversal, remaining negative at neutral pH but turning positive in endosomes (pH ∼5.0) to enable membrane-disruption-mediated escape. Furthermore, assembling iFTn into chain-like tetramers (iFTn-4er) via a two-armed PEG crosslinker synergistically enhances both cellular uptake and endo-/lysosomal escape compared to simpler assemblies (Figure 6). Another cutting-edge strategy utilizes engineered bioactive peptides to synergistically enhance cellular penetration and endosomal escape. For example, fusing the cell-penetrating peptide TAT with the membrane penetration-enhancing peptide S19 for siRNA delivery: TAT mediates cellular uptake, while the S19 peptide promotes TAT dimerization within late endosomes to effectively drive escape and enable efficient, low-toxicity cytosolic delivery. Initially, this peptide pair was fused to an RNA-binding domain (RBD); however, while escape improved, excessively tight siRNA binding hindered cytoplasmic release and loading into the RNA-induced silencing complex (RISC), limiting silencing efficacy. To resolve this, the researchers directly conjugated TAT-S19 to the RISC core protein Ago2, creating a pre-assembled TAT-S19-Ago2/siRNA complex. This design delivers a pre-assembled, bioactive “minimal RISC” directly into the cytosol. Upon endosomal escape, the complex is immediately functional, enabling rapid and potent gene knockdown without requiring cytosolic reassembly [154] (Figure 7).

### Clearance by the cardiac immune system

**Figure 8:**
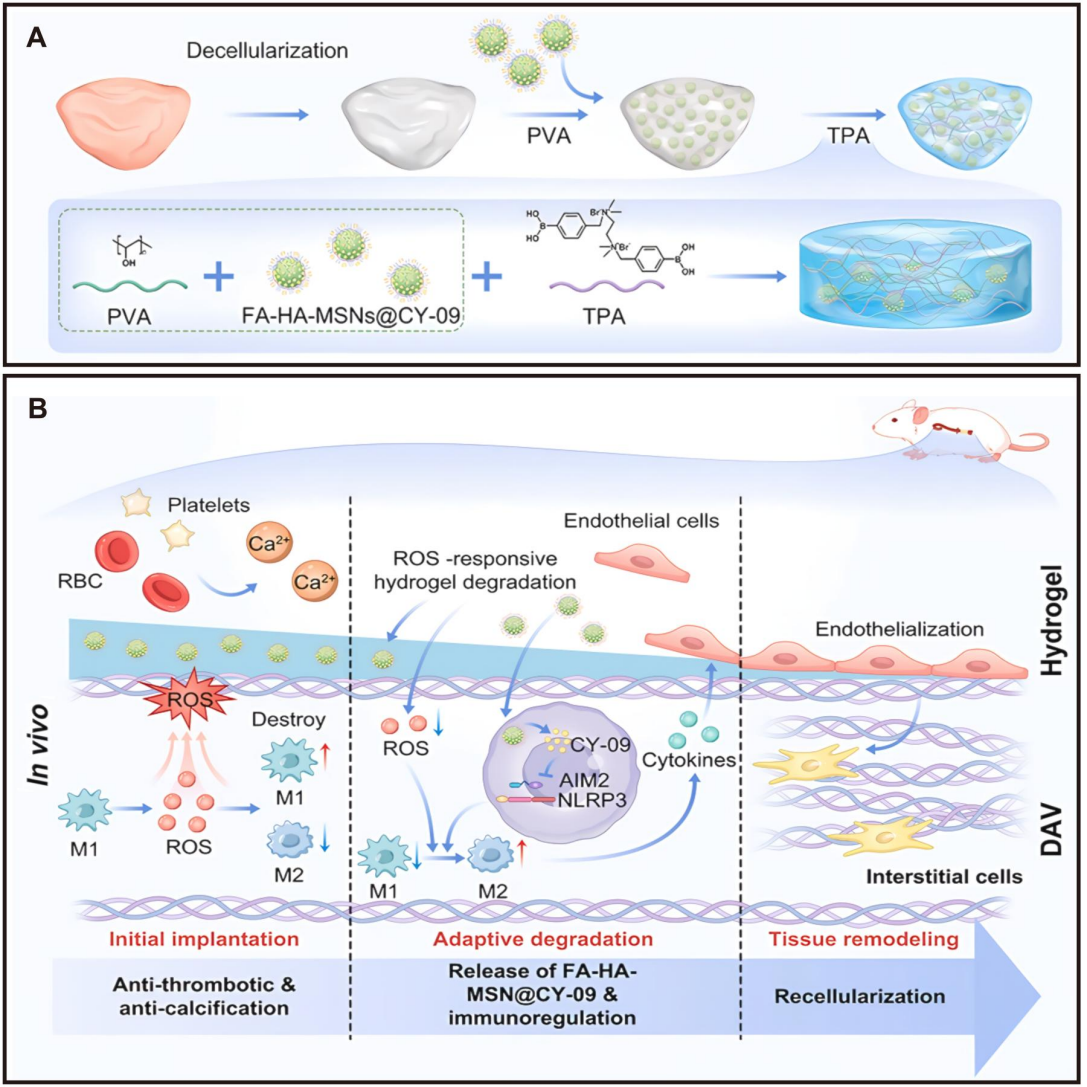
Immunomodulation of macrophages via a ROS-responsive nanoreservoir system for enhanced cardiac tissue engineering graft repair. (**A**) Composition of the DAVs/ROS-Gel@MSNs scaffold. (**B**) Schematic illustration of the immunoregulatory and regenerative mechanism of DAVs/ROS-Gel@MSNs scaffold [158]. Copyright 2025, Elsevier.

Cardiac tissue harbors resident macrophages that are independent of monocyte recruitment from the circulation. These cells monitor tissue homeostasis and clear foreign substances. A significant portion of intravenously injected nanocarriers is recognized and phagocytosed by these immune cells, effectively removing them from the target area [155]. Under inflammatory conditions post-MI, the phagocytic activity of macrophages is further enhanced, leading to the clearance of many drugs before they can take effect [155]. To counteract this immune clearance, efforts can focus on modulating macrophage functional states [156]. For instance, targetedly designed monocyte chemoattractant protein-1 (MCP-1) binding peptides can neutralize MCP-1, preventing the migration of cardiac resident macrophages. Alternatively, N-acetylneuraminic acid can mimic host cell surface characteristics to evade immune system recognition. These methods have been proven effective in reducing nanoparticle clearance rates [155, 157]. Similarly, tissue-engineered cardiac grafts often trigger immune responses affecting cardiac tissue remodeling. Loading drugs capable of reprogramming macrophage function has been shown to effectively suppress inflammation, promote a regenerative microenvironment and drive tissue repair [158] (Figure 8).

## Conclusion and future perspectives

Mitochondrial dysfunction in CVDs, as a central pathological hub, presents an attractive target for developing novel therapeutic strategies. This review systematically outlines the key roles of mitochondria in major CVDs like AS, MIRI and HF and focuses on the design strategies, influencing factors and application progress of nanocarriers aimed at precisely regulating mitochondrial function.

Current research indicates that through sophisticated nano-engineering, hierarchical targeting of diseased cardiac tissue and its internal mitochondria is achievable. This involves utilizing carriers with good biocompatibility, precisely tuning nanoparticle size, morphology and surface properties to optimize pharmacokinetics, employing cardiac or disease-specific targeting peptides to enhance enrichment at the lesion site and ultimately using mitochondria-targeting ligands to guide therapeutics to the final target—the mitochondria. These strategies have shown promising potential in preclinical models, achieving initial success particularly in mitigating oxidative stress damage in MIRI, improving energy metabolism in HF, and promoting myocardial tissue repair, indicating high clinical translation value.

**Figure 9:**
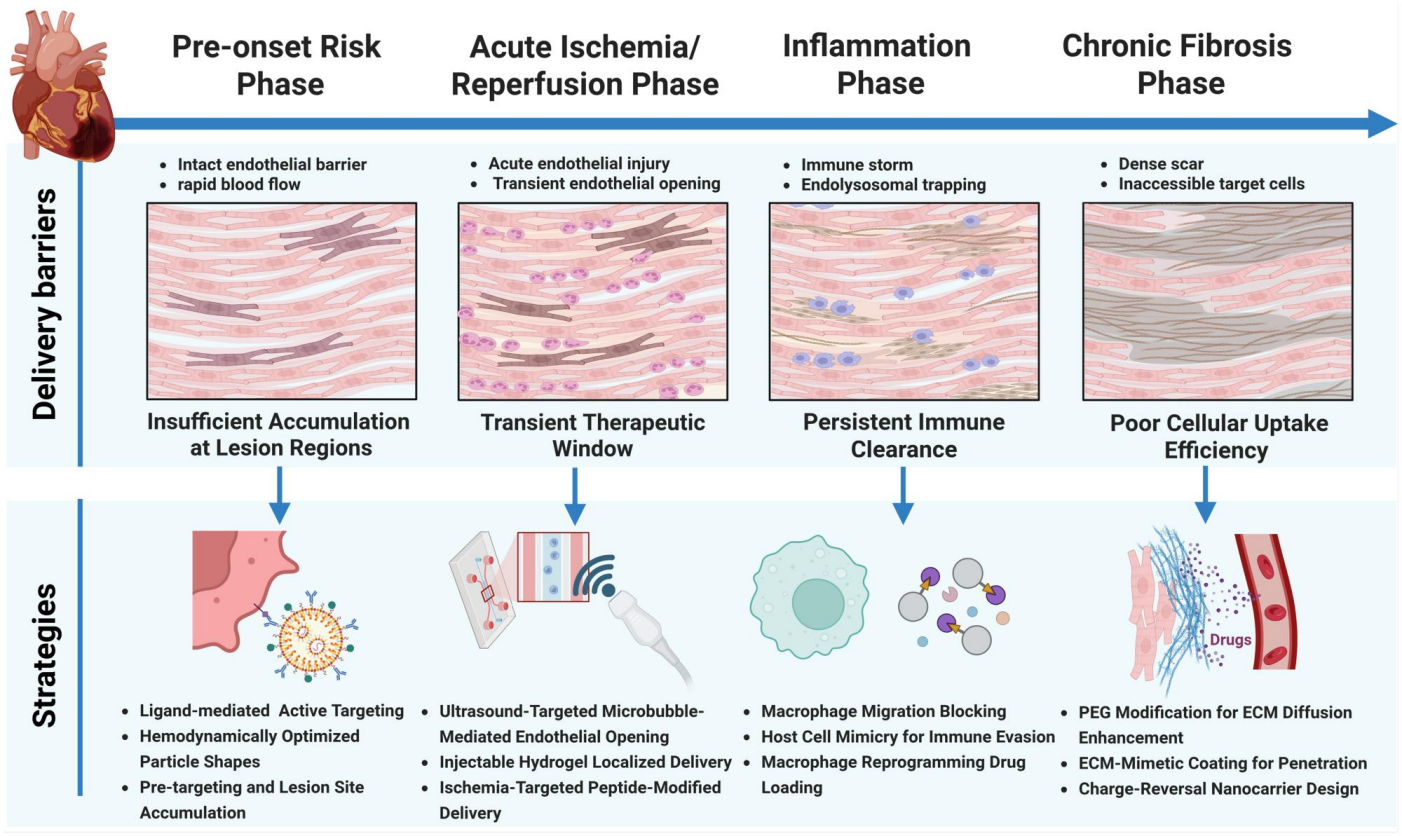
Delivery barriers and targeted strategies across myocardial infarction progression phases. Schematic illustration of targeted nanomedicine strategies to overcome delivery barriers across the four sequential phases of myocardial infarction. (Image created with BioRender.com, with permission).

However, it must be clearly recognized that translating mitochondria-targeted nanomedicines into clinical therapies for CVDs still faces a long journey. Currently, the field is largely in the preliminary stages of preclinical exploration and proof-of-concept studies, with effective delivery and long-term safety being the main challenges. From overcoming high-velocity cardiac blood flow and endothelial barriers following systemic administration, to being effectively internalized by poorly endocytic cardiomyocytes, to successfully escaping lysosomal degradation and ultimately reaching functionally impaired mitochondria, every step in the delivery chain requires efficiency improvements. Despite substantial advances in the mechanistic understanding of various mitochondria-targeting ligands, their translation into broadly effective therapeutic tools remains challenging. A critical determinant of therapeutic success lies in the alignment between the ligand’s mechanism of action and the complex pathophysiological context of the disease. Key factors such as the acute or chronic stage of the illness, as well as patient-specific genetic backgrounds that influence protein interactions, significantly inform the selection of an appropriate mitochondrial targeting strategy (Figure 9). Regarding long-term safety, a systematic risk assessment for mitochondrial-targeted nanotherapeutic platforms is urgently required. First, the chronic bioaccumulation of nanocarriers in organs such as the liver and kidneys and the mechanisms underlying their chronic toxicity remain poorly understood. Second, their prolonged retention in cardiac tissues or mitochondria may disrupt normal energy metabolism and ion homeostasis; certain targeting ligands, due to persistent accumulation, could disturb mitochondrial membrane potential or even inhibit respiratory chain function. Moreover, the long-term presence of nanocarriers may activate innate or adaptive immune responses, leading to chronic inflammation or autoimmunity. Therefore, developing nanocarriers that balance targeting efficiency with biodegradability, and establishing a comprehensive long-term evaluation system covering mitochondrial function, physiological signaling pathways, immune responses and multi-organ toxicology, represent critical prerequisites for advancing the clinical translation of this field. Furthermore, the complexity of the cardiac microenvironment and individual differences impose extremely high demands on the rational design of nanomedicines. Nanotherapeutics must move beyond the broad-spectrum delivery approach and instead adopt a strategy guided by the mechanism of drug action for precise patient stratification. This involves optimizing nanocarriers to enhance drug bioavailability and dosing rationality, while integrating diagnostic and therapeutic modules to enable accurate efficacy evaluation [159–161].

Future advancements in this field will likely depend on deeper integration of multiple disciplines. Progress in materials science will give rise to smarter, more biocompatible responsive carriers. A deeper understanding of cardiovascular pathobiology and nano-bio interactions will guide the design of more precise targeting strategies [162, 163]. Close collaboration between pharmacology and clinical medicine is key to bridging the gap from animal models to human application. Current research predominantly focuses on antioxidant stress therapy targeting mitochondria. However, clinical translation experience indicates that intervention strategies aimed solely at scavenging ROS exhibit significant limitations, often failing to adequately address the dysregulation of complex molecular networks that drive disease progression [164]. Actually, ROS act not merely as toxic byproducts but as crucial signaling molecules. Their indiscriminate removal may disrupt physiological processes [165]. Moreover, the pathological rise in ROS is often a downstream consequence of more fundamental mitochondrial dysfunction, such as impaired quality control or metabolic inflexibility. Therefore, treating only the “smoke” (ROS) without addressing the “fire” (core functional deficits) yields incomplete therapeutic outcomes [166]. Future research directions may shift toward exploring other aspects of mitochondrial-targeted therapies. This includes novel approaches to modulate mitophagy, biogenesis and metabolic flexibility, as well as emerging applications in delivering genetic material for mitochondrial gene therapy. For example, CRISPR/Cas9-based gene editing has been successfully utilized to construct reporter systems for dynamically assessing mitophagy in human cardiac tissues and for screening key kinases regulating this process [167]. Concurrently, the modulation of critical mitochondrial biogenesis pathways by agents like tetrahydrobiopterin, coenzyme Q10 and calycosin is emerging as a potential therapeutic strategy [168–170]. The small molecule activator sodium butyrate, which regulates the AMPK-PGC-1α axis to optimize fatty acid oxidation and glucose metabolism, has demonstrated potential in improving mitochondrial adaptability in models of obesity and diabetes [171]. In terms of delivery technology, multistage responsive nanocarriers enable tissue targeting, lysosomal escape and precise mitochondrial localization, offering a practical platform for the efficient and low-toxicity delivery of mitochondrial gene-editing tools [172].

Furthermore, combining mitochondrial imaging technologies will aid in real-time visualization of mitochondrial protection effects, elevating the level of precision therapy. It should be further noted that the current therapeutic paradigm of mitochondria-targeted nanomedicines primarily focuses on “cytoprotection”—that is, rescuing damaged cardiomyocytes through strategies such as alleviating oxidative stress and restoring energy homeostasis, with the aim of preserving existing tissue function to the greatest extent possible. However, to achieve fundamental restoration of cardiac structure and function following cardiovascular diseases, particularly myocardial infarction, an ideal objective lies in promoting “cardiac regeneration,” which entails the supplementation and effective replacement of functional cardiomyocytes. This involves a complex process requiring the coordinated activation of multiple pathways, including cell cycle re-entry, extracellular matrix remodeling and angiogenesis. Consequently, a central evolutionary direction for future research is to design a new generation of mitochondria-targeted nanoplatforms that not only enable efficient cytoprotection but also encode and deliver regenerative signals, thereby synergizing with or initiating endogenous repair programs to advance therapeutic outcomes from “cellular salvage” to “tissue reconstruction.” This demands that nanomedicine research not only focus on delivery efficiency and targeting precision but also delve deeper into exploring how to achieve temporally and combinatorially controlled integration of mitochondrial function repair with the biological pathways underlying myocardial regeneration.
